# Microbial Diversity and Organic Acid Production of Guinea Pig Faecal Samples

**DOI:** 10.1007/s00284-019-01630-x

**Published:** 2019-02-12

**Authors:** Susakul Palakawong Na Ayudthaya, Hans van der Oost, John van der Oost, Daan M. van Vliet, Caroline M. Plugge

**Affiliations:** 1 Laboratory of Microbiology, Wageningen University & Research, Stippeneng 4, 6708 WE Wageningen, The Netherlands; 2 Biodiversity Research Centre, Thailand Institute of Scientific and Technological Research, 35 Mu 3 Technopolis, Khlong Ha, Khlong Luang, Pathumthani, 12110 Thailand

## Abstract

The guinea pig (*Cavia porcellus*) or cavy is a grass-eating rodent. Its main diet consists of grass or hay, which comprises cellulose, hemicellulose, lignin and their derivatives. Here, the microbial diversity of faecal samples of two guinea pigs and microbial enrichments made with substrates, including starch waste and dried grass, were investigated along with organic acid production profiles. The microbial communities of the faecal samples were dominated by the phyla Bacteroidetes (40%) and Firmicutes (36%). Bacteroidales S24-7 (11% in Cavy 1 and 21% in Cavy 2) was the most abundant order. At genus level, many microorganisms remained unclassified. Different carbon sources were used for organic acid production in faecal enrichments. The dominant bacterial groups in the secondary enrichments with dried grass, starch waste and xylose were closely related to *Prevotella* and *Blautia*. Acetate was the predominant organic acid from all enrichments. The organic acid production profiles corresponded to a mixed acid fermentation but differed depending on the substrate. Eight phylogenetically different isolates were obtained, including a novel *Streptococcus* species, strain Cavy grass 6. This strain had a low abundance (1%) in one of the faecal samples but was enriched in the dried grass enrichment (3%). Cavy grass 6, a fast-growing heterolactic bacterium, ferments cellobiose to lactate, acetate, formate and ethanol. Our results show that cavy faecal samples can be applied as microbial source for organic acid production from complex organic substrates. The cavy gut contains many as-yet-uncultivated bacteria which may be appropriate targets for future studies.

**Electronic supplementary material:**

The online version of this article (10.1007/s00284-019-01630-x) contains supplementary material, which is available to authorized users.

## Introduction

Guinea pigs (*Cavia porcellus*) are rodents belonging to the family *Caviidae* and are native to South America [32]. They are well known as experimental models for humans and have been used in medical research since the nineteenth century [12]. In gastrointestinal research, guinea pigs are considered suitable models for humans because they have human-like E-cadherin on their intestinal surface [12]. To date, limited studies have been performed on the microbial composition of the guinea pig gut. In 2012, the intestinal microbiota of guinea pigs was studied using a metagenomic approach, revealing a higher abundance of *Akkermansia* spp. and methanogens (*Methanobrevibacter* spp.) compared to the human gut [12]. Recently, the microbial population from domesticated guinea pigs and rabbits was compared [5]. Differences were detected between samples from rabbit and guinea pig faeces, suggesting that there is no a microbial community common in coprophagous (faeces-eating) animals. Those animals eat their own faeces to maintain their intestinal microbes and recover nutrients and vitamins [31]. *Bacteroidetes* and *Firmicutes* together formed most of the population in the guinea pig faecal samples, according to the results of two studies [5, 12]. The two most abundant bacterial phyla in guinea pig guts relate to the typical vertebrate gut microbiome, including the human intestine. However, at genus level, the microbiome composition was different between humans and guinea pigs [12].

The guinea pig is a monogastric herbivore [32] and its diet mainly consists of grass or hay of timothy, oat, wheat, pasture, meadow and/or ryegrass. Grass-eating rumen mammals such as cows and sheep have a long digestive tract or diet re-chewing system to digest and obtain nutrients, whereas guinea pigs have a relatively short digestive tract, therefore they maintain their vitamins and nutrition by re-eating their own faeces. Consuming large quantities of plant polymers suggests that its gastrointestinal microbiome generates (hemi-)cellulolytic enzymes. The corresponding microbes may be useful for biotechnological applications.

The most abundant sources of organic carbon in global ecosystems are complex polysaccharides of plant cell walls that are difficult to degrade [18]. Cellulose degradation is usually driven by complex microbial communities such as bacteria and fungi, which use cellulolytic enzymes, 1,4-β-endoglucanase, 1,4-β-exoglucanase and/or β-glucosidase that hydrolyse cellulose to cellobiose and/or to glucose, which can then be further metabolized [16]. The microorganisms involved in cellulose degradation from the cavy gut are understudied [18, 38].

Organic acids (OAs) such as acetate, lactate and succinate are common fermentation products of plant polysaccharides. OAs can be used as biobased building-block chemicals in chemical and other industrial processes; therefore, the production of chemicals from renewable resources is considered an attractive green alternative [1, 34]. Investigating the microbial diversity and organic acid production of guinea pig faecal samples could also be instrumental in revealing the mechanism of the guinea pig fibre digestion system and may lead to the discovery of novel bacteria capable of converting cellulose to organic acids. To date, no research has been performed using guinea pig faecal samples as a source for OA production.

In this study, the microbial diversity of guinea pig faecal samples was analysed. OA production profiles from cellulose, dried grass, glucose, starch waste, xylan and xylose by guinea pig faecal enrichment cultures were studied. Moreover, the microbial community composition of selected enrichments was revealed, and several pure cultures were isolated.

## Materials and Methods

### Sampling, Screening and Enrichment of Cavy Faecal Samples with Various Substrates

A schematic overview of the experiments performed in this study is shown in Fig. S1.

### Source of Inoculum

Fresh faecal droppings were collected from two adult-male pet guinea pigs (four and 3 years old; Cavy 1 and Cavy 2), in Renkum, The Netherlands. The faecal samples were placed into anaerobic sterile bottles and directly transferred to the Laboratory of Microbiology (Wageningen University & Research, Wageningen, NL), where the entire experiment was performed. Approximately 1 g of the faecal samples was dissolved in anaerobic phosphate-buffered saline (PBS) using a sterile spatula and gentle vortexing in an anaerobic chamber. The guinea pig faecal slurry (1% v/v) was then used as an inoculum for enrichment with various substrates.

### Substrate Preparation

Cellulose, dried grass (commercial cavy grass feeding), glucose, soluble starch, starch waste, xylan and xylose (0.5% w/v) were used as carbon sources to selectively enrich bacteria from guinea pig faeces. The dried grass was cut into small pieces using scissors (1–3 mm). The starch waste (80% dry matter and 20% water) was prepared and analysed as previously described [27].

### Medium Composition and Cultivation

A bicarbonate-buffered anaerobic medium (BM) used in this experiment was prepared and supplemented as previously described [26, 28]. Then, 0.5% (w/v) of each substrate was added into serum bottles (duplicate bottles for each enrichment) before autoclaving. Bottles without substrate were used as controls.

One percent (v/v) (pooled faecal samples) was inoculated in the anaerobic bottles and bottles were incubated at 37 °C in the dark for 14 days. Growth, pH and OA production of the enrichments were measured to determine microbial activity. The primary enrichments were transferred (4% v/v) to medium with the same substrate as previously described and termed secondary enrichments. After 5 days of incubation at 37 °C, the secondary enrichments were used for further analysis.

### DNA Extraction

Genomic DNA from fresh faeces of each guinea pig, from selected secondary enrichment samples and the isolates was extracted using FastDNA® SPIN Kit for Soil (MP Biomedicals; Santa Ana, CA) following the manufacturer’s instructions. Genomic DNA yields were measured with a Nanodrop ND-1000 spectrophotometer (Nanodrop Technologies, Wilmington, DE). DNA quality was analysed using OD 260/280 ratio and the integrity was determined by gel electrophoresis on a 1% (w/v) agarose gel. The extracted genomic DNA was then kept at −20 °C for further analyses.

### 16S rRNA Amplicon Sequencing Analysis

The extracted genomic DNA was diluted to obtain DNA concentration between 10 and 20 ng/µl as a template for PCR amplification. Microbial 16S rRNA V4 regions were amplified using a two-step PCR protocol. PCR amplifications were carried out in technical duplicates. The first PCR was performed with universal primers 515f (5′-GTGCCAGCMGCCGCGGTAA-3′) and 806r (5′-GGACTACHVGGGTWTCTAAT-3′) [2] and the second PCR was carried out to extend eight-base specific barcodes to the amplicons as previously described [10] using Phusion Hot Start II High-Fidelity DNA polymerase (Thermo Fisher Scientific; Waltham, MA). PCR amplification was performed using a G-Storm cycler (G-storm; Essex, UK). The first PCR was performed in a total volume of 50 µl containing 2.5 µl of each forward and reverse primer, 0.5 µl (2 units) of the DNA polymerase, 10 µl of 5 × HF-buffer, 1 µl (200 µM) dNTP mix, 1 µl of DNA template and 32.5 µl of nuclease-free sterile water using the PCR program as follows: a pre-denaturing step at 98 °C for 3 min, followed by 25 cycles at 98 °C for 10 s, 50 °C for 20 s, 72 °C for 20 s and a post-elongation step of 10 min at 72 °C. After amplification, the second PCR was done in 100 µl containing 10 µl of the barcoded primer mix, 1 µl (2 units) of the DNA polymerase, 20 µl of 5 × HF-buffer, 2 µl (200 µM) dNTP mix, 5 µl of DNA template and 62 µl of nuclease-free sterile water with the PCR program as follows: a pre-denaturing step at 98 °C for 30 s, followed by 5 cycles at 98 °C for 10 s, 52 °C for 20 s, 72 °C for 20 s and a post-elongation step of 10 min at 72 °C. The size of PCR products was expected to be 291 bp. Barcoded PCR products were examined for positive amplification on agarose gel and were then purified using the CleanPCR kit system according to the manufacturer’s instruction (CleanNA Alphen aan den Rijn, The Netherlands). The DNA concentration was quantified using Qubit® dsDNA BR Assay Kit (Invitrogen) and DeNovix DS-11 FX Spectrophotometer/Fluorometer (DENovix Inc.; Wilmington, DE). All purified PCR products were pooled in equimolar amounts (200 ng of DNA per sample) to create a library which was then purified again with the CleanPCR kit to a final volume of 35 µl. The library was sent for paired-end Illumina MiSeq sequencing at GATC Biotech (Konstanz, Germany).

16S rRNA gene MiSeq sequencing data were analysed with NG-Tax version 1.0 [30] using default settings apart from a read length of 200 bp and a 93% identity threshold for taxonomic assignment (‘error correction’ in NG-Tax). Paired-end libraries were filtered to obtain only read pairs with perfectly matching barcodes and those barcodes were then used to demultiplex reads by samples. Taxonomic assignment was performed with the SILVA 16S rRNA reference database (release version 128) using an open reference approach as described by Quast et al. [29].

### Denaturing Gradient Gel Electrophoresis (DGGE) Analysis

DGGE was used to compare secondary enrichments. Bacterial 16S rRNA V6–V8 regions were amplified with the DGGE Universal primers GC-968F (5′-CGCCCGGGGCGC GCCCCGGGCGGGGCGGGGGCACGGGGGGAACGCGAAGAACCTTAC-3′) and 1401R (5′-CGGTGTGTACAAGACCC-3′) [24] using the Phire Hot Start II High-Fidelity DNA Polymerase (Thermo Fisher Scientific; Waltham, MA). Bacterial amplicons were produced with a G-Storm cycler (G-storm; Essex, UK) using a pre-denaturing step at 95 °C for 5 min, followed by 35 cycles at 95 °C for 20 s, 56 °C for 40 s, 72 °C for 40 s and a post-elongation step of 10 min at 72 °C. The forward primer had a GC clamp of 40 bp attached to the 5′ end as used by Yu et al. [41]. DGGE analysis was performed as described previously [20] using a DCode TM system (Bio-Rad Laboratories; Hercules, CA) at 60 °C for 16 h with a denaturing gradient of 30:60 percent for bacterial profiles [41].

### Clone Library Construction

The secondary enrichments (dried grass, starch waste and xylose) were further analysed using a clone library approach. Almost full-length 16S rRNA genes were amplified using bacterial-universal primers 27f (5′-AGAGTTTGATCCTGGCTCAG-3′) and 1492r (5′-TACCTTGTTACGACTT-3′) [17]. PCR amplification was performed with the GoTaq Polymerase kit (Promega; Madison, WI) using a G-Storm cycler (G-storm; Essex, UK). The PCR program was started with a denaturing step at 95 °C for 5 min and continued with 35 cycles consisting of 95 °C for 30 s, 52 °C for 40 s and 72 °C for 90 s and the last step of extension at 72 °C for 7 min. PCR products were purified using the PCR Clean & Concentrator kit (Zymo Research Corporation; Irvine, CA). Amplicons were ligated into a pGEM-T Easy vector kit (Promega; Madison, WI) and transformed into *E. coli* XL1-Blue Competent Cells (Agilent Technologies; Santa Clara, CA). Both ligation and transformation were conducted according to the manufacturer’s instruction following the blue–white screening technique. White colonies were randomly selected and transferred to a 96-well Masterblock plate (Greiner Bio-One; Netherlands). 16S rRNA genes were sequenced using primer SP6 (5′-ATTTAGGTGACACTATAG-3′) (Promega Corp.; Madison, WI) at GATC Biotech (Konstanz, Germany). DNA sequences were trimmed by removing the primer using the program DNA Baser Sequence Assembler v4 (Heracle BioSoft S.R.L; Arges, Romania). Chimeras were identified using DECIPHER’s Find Chimeras web tool [39] and were removed. The 16S rRNA sequences were blasted with the NCBI online database.

### Isolation and Identification of Fast-Growing Bacteria

Each of the secondary enrichment was diluted 10-fold using liquid Reinforced Clostridial Medium (RCM) and plated on modified BMY (BM with 0.1 g l^−1^ yeast extract), supplemented with 5 mg l^−1^ hemin, 0.05 g l^−1^ vitamin K_1_, 0.5 g l^−1^l-cysteine-hydrochloride and 15 g l^−1^ agar (Difco) and the same carbon source as the original enrichment. The plates were incubated under anaerobic conditions with N_2_–H_2_ (96:4 v/v) gas at 37 °C for 5 days. Single colonies were picked and further purified on the same agar medium by the streak plate method, followed by serial dilution in the modified BMY liquid medium with the same substrate as described above for three times to obtain pure cultures. The pure cultures were grown in anaerobic bottles with BMY media with 20 mM glucose and analysed routinely by phase-contrast microscopy (Leica DM 2000; Wetzlar, Germany).

Genomic DNA of the isolated strains was amplified to obtain almost full-length 16S rRNA gene sequence using the same PCR primers and PCR protocol as previously described in the clone library analysis or by Palakawong Na Ayudthaya et al. [25]. 16S rRNA gene sequencing was performed at GATC Biotech (Konstanz, Germany). The 16S rRNA gene sequences were checked for reading errors and aligned using the program DNA Baser Sequence Assembler v4 (Heracle BioSoft S.R.L; Arges, Romania) and were then searched against the NCBI database using the BLASTN search online program (http://blast.ncbi.nlm.nih.gov/Blast.cgi: 21-12-2016).

### Analytical Methods

Growth was monitored by measuring the turbidity at 600 nm using a spectrophotometer for enrichments with soluble substrates (glucose and xylose). The organic acid production was measured by high-pressure liquid chromatography (HPLC) as previously described [36]. Methane and hydrogen were quantified using gas chromatography (GC) as described by Van Lingen et al. [37].

### Nucleotide Sequence Accession Numbers

The 16S rRNA gene MiSeq sequences from the cavy faecal samples and the secondary enrichments and the 16S rRNA gene sequences of clone library from the secondary enrichments were deposited at the EMBL database and are available under accession numbers ERS1974899–ERS1974908 (PRJEB21993) and LT708382–LT708474, respectively. The bacterial 16S rRNA gene sequences of the isolates were deposited to NCBI and EMBL databases and are available under accession numbers MF579703–MF579713 and LT546457, respectively.

## Results and Discussion

### Microbial Community Composition of Guinea Pig Faecal Samples

In total, 536,464 high-quality sequences were obtained from the fresh faecal samples of ‘Cavy 1’ and ‘Cavy 2’, which clustered into 78 operational taxonomic units (OTUs) at genus level. Of those 78 OTUs, 34 were shared between both animals and 44 were unique OTUs, distributed over 14 OTUs in Cavy 1 and 30 OTUs in Cavy 2. Numbers of OTUs for each sample at phylum level are shown in Table S1.

**Fig. 1:**
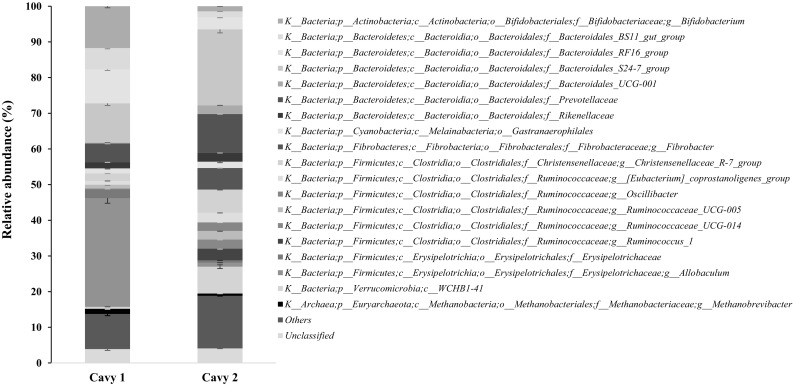
Relative abundance of microorganisms in two guinea pig faecal samples, at genus level. Phylogenetic groups that contributed less than 1% in both samples are in category “others”. Sequences that could not be not be assigned at six different identity-threshold levels are grouped as “Unclassified”. Error bars give variation between technical duplicates

The phyla Firmicutes (42% and 31%), Bacteroidetes (35% and 45%), Actinobacteria (13% and 2%) and Verrucomicrobia (1% and 9%) had a high relative abundance in both faecal samples (Cavy 1 and 2, respectively), whereas Fibrobacteres, Cyanobacteria and Spirochaetae represented together around 11% in both samples. Our results are in agreement with previous work, though the relative abundance of the phyla was different [5]. Euryarchaeota were detected in both samples (1.5 and 0.6% in Cavy 1 and Cavy 2, respectively) (Fig. 1). The 16S rRNA gene sequence of this archaeon OTU showed 99% identity to *Methanobrevibacter smithii*, a dominant archaeon in the human gut [33].

At family level (in both Cavy 1 and 2), Bacteroidales S24-7 group (11% and 21%, respectively) was most abundant, followed by Prevotellaceae (5% and 11%, respectively) (Fig. 1). In Cavy 1, the genus *Allobaculum* had a very high relative abundance (31%), while only 1% was detected in the sample from the Cavy 2. *Allobaculum* spp. was previously identified as the most active glucose utiliser in mice [11]. About 5.7% relative abundance of *Allobaculum* was previously found in guinea pig gut [22] and was also detected in other animals’ guts, including in dogs [9], mice and hamsters [21]. *Bifidobacterium*, a probiotic species in the phylum Actinobacteria that is believed to be important to the host health, was highly abundant (12% relative abundance) in Cavy 1 but not in Cavy 2 (1% relative abundance). On the other hand, *Fibrobacter*, a fibre-degrading species, and *Ruminococcus* were found in Cavy 2 with relative abundances of 6% and 3%, respectively, and were not detected in Cavy 1. Crowley et al. [5] similarly reported that the genus *Fibrobacter* was detected in high abundance (11%) in only one of six guinea pigs. The researchers suggested that in the cavy gut, organisms other than the *Fibrobacteres* must be responsible for fibre digestion [5].

The results of both faecal samples suggested that each guinea pig has its own microbiome even when living together and eating the same diet. Nguyen et al. [23] also reported that only 13% of the genera were shared in three murine datasets. Moreover, 57% and 78% from both cavy faecal samples could not be assigned at the genus level (Fig. 1). In all cavy microbiome studies, including ours, a substantial percentage of the population remains unclassified at the genus level, indicative of novel biodiversity [22].

### Product Profiles

**Fig. 2:**
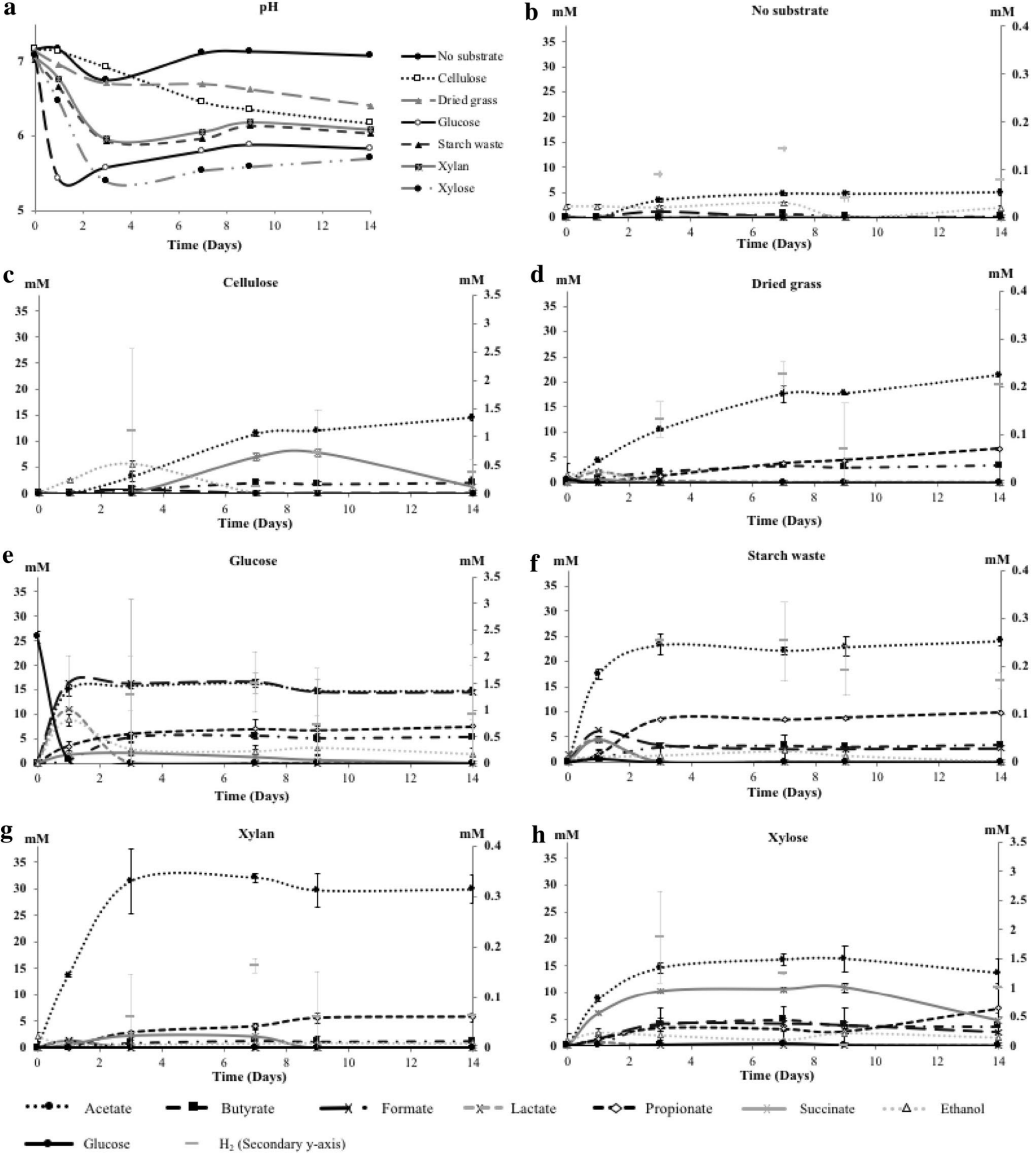
pH profile of all fermentations in **a**. Guinea pig faecal slurry fermentation profiles from different carbon sources of the primary enrichments incubated at 37 °C for 14 days (**b**–**h**). The error bars indicate the standard deviation

In all primary enrichments, mixed acid fermentation to acetate, butyrate, lactate, propionate, succinate and ethanol occurred with cellulose, dried grass, glucose, starch waste, xylan and xylose (Fig. 2). Glucose was degraded fastest (Fig. 2e) with simultaneous acidification (Fig. 2a), followed by starch waste, xylose, xylan, cellulose and dried grass (Fig. 2). The highest total OA production was reached at different time points depending on the substrate type (Fig. 2 and Table S2). Fermentation of cellulose and dried grass was slower with total OA production of 22 mM and 31 mM at day 9 and day 14, respectively. In general, acetate was the main product in all the enrichments. The highest succinate concentration (11 mM) was measured in the xylose enrichment (Fig. 2b–h and Table S2). Lactate was mainly formed during glucose and starch waste enrichments (11 and 5 mM, respectively). OA concentration decreased by the end of the fermentation period, particularly in the case of succinate. In primary enrichments, a dynamic fermentation was on going (Fig. 2c, e, f, g and h and Table S2).

In the secondary enrichments, mixed acid fermentation again occurred, and acetate, butyrate and propionate were produced with all substrates (dried grass, starch waste and xylose). Formate, lactate, succinate and/or ethanol were produced in some substrates, indicating that different microbial communities developed (Table S3).

Taken together, the different substrates yielded different mixed acid profiles, with acetate as the main product. All complex substrates (cellulose, dried grass and starch waste) were fermented by the microbial community from guinea pig faecal samples. Based on the OA production profiles, the succinate production and the efficiency of the substrate utilization, dried grass, starch waste and xylose enrichments were selected for further analysis.

### Microbial Community Profiling of Secondary Enrichments

#### Major Groups Detected

Bacterial DGGE profiles of guinea pig faecal samples from the secondary enrichments of dried grass, starch waste and xylose are shown in Fig. S2. Based on the profiles, three bacterial 16S rRNA gene clone libraries were constructed. Additionally, a microbial 16S rRNA gene MiSeq analysis was used to investigate the relative abundance of the microorganisms from the secondary enrichments. The majority of the clones were distantly related to cultured relatives and may represent novel genera or species, according to the respective 16S rRNA gene sequence identity thresholds of 94.5% [40] and 98.7% [35], respectively (Table S4). About 60% of all clones had less than 94% identity to their closest cultured relative (Table S4), again indicative of the presence of multiple novel genera in the cavy gut.

The 16S rRNA gene MiSeq results revealed highest bacterial diversity in the dried grass enrichment while the lowest was detected in the xylose enrichment. These results were supported by the number of bands in the DGGE gel profiling (Fig. S2). Firmicutes were the most abundance in the dried grass enrichment with 67%, whereas Bacteroidetes were the most abundance in the starch waste and xylose enrichments (61% and 64%, respectively). Actinobacteria were detected in all enrichments with a low abundance (3% on average).

**Fig. 3:**
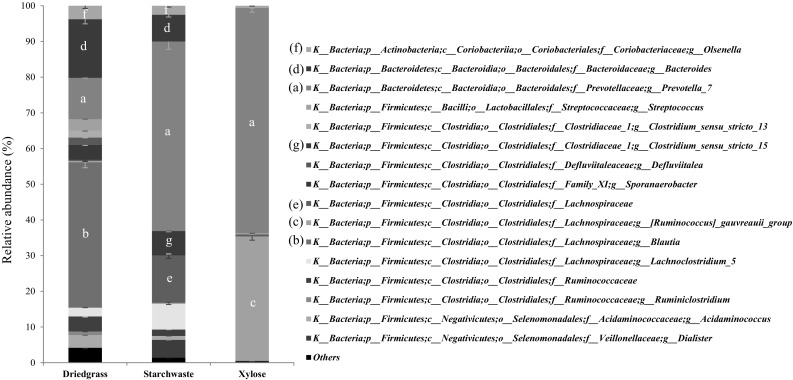
Relative abundance of taxa in the 16S rRNA MiSeq dataset of the secondary enrichments, at genus level. Phylogenetic groups that contributed less than 1% in both samples are in category “others”. Error bars give variation between technical duplicates. The major genus-level phylogenetic groupings from all samples were alphabetically emphasized as a–g

Comparing the two approaches (clone library and MiSeq sequencing analyses), slightly different compositions were found, which could be due to the different primers used and to the small size of [7, 15]. Moreover, the different lengths of the amplified sequences from both methods resulted in varying % identification comparing to the database (200 bp for MiSeq and ~720 bp for the clone library). Both approaches detected *Prevotella* spp. in all enrichments (Table S4, Fig. 3). However, the clones were only distantly related to *Prevotella* spp. (≤95%) and represent novel. Both approaches revealed *Prevotella* as being the core of the microbial community in the secondary enrichments. Therefore, the genus *Prevotella* may play an important role in fibre degradation in the guinea pig gut. Members of the genus *Prevotella*, known to carry genes for cellulose and xylan hydrolysis [13], can efficiently convert xylan, xylose and/or carboxymethylcellulose into short-chain fatty acids [6]. *Prevotella* enterotypes were also dominant in the gut of mice and humans that had carbohydrate- and fibre-rich diets [6, 23]. *Prevotella* spp. have also been reported to show a positive correlation with xylose in pig guts and it is known as an acetate-producing bacterium [13]. The Genus *Prevotella* is known as a dietary fibre-fermenting bacterium [4] and recently it was shown that it became dominant in the calve gut upon a change to fibre diets [14].

*Blautia* was the most abundant OTU (41% relative abundance) in the dried grass enrichment, while less than 1% was detected in the starch waste enrichment and it was not detected at all in the xylose enrichment (Fig. 3). However, *Blautia* was detected in all clone libraries of the three enrichments (dried grass, starch waste and xylose) with high relative abundance (Table S4). Acetate is the main fermentation product of *Blautia producta* (formerly *Ruminococcus productus)* [19]. In our study, *B. producta*-related clones (96–98% identity) were abundant in the dried grass (11%), starch waste (14%) and xylose (5%) enrichments (Table S4).

The second most abundant group in the dried grass enrichment by MiSeq sequencing analysis belonged to genus *Bacteroides* (16%). *Bacteroides* was also present in the enrichment of starch waste (8%) but was not detected in the xylose enrichments. *Bacteroides*-related clones were detected in all enrichments. Clone sequences related to *Bacteroides xylanisolvens* (99% 16S rRNA gene sequence identity) were detected in the enrichments of dried grass and starch waste with 6 and 3% relative abundances, respectively, and the clone sequences related to *B. xylanisolvens* (90% 16S rRNA gene sequence identity) were also detected with 5% relative abundance in the xylose enrichment. *B. xylanisolvens* is known to degrade xylan and xylose to mainly acetate, propionate and succinate, but cannot utilize starch [3]. The starch waste contains various compounds such as sugar, protein and/or other substances that bacteria can use for growth, as well as the benefit of mixed cultures in the enrichment that the bacteria can share their products. The 90–99% 16S rRNA gene sequence identity points to the possibility of a different *Bacteroides* strain.

The second most abundant bacteria detected in the xylose enrichment by MiSeq sequencing analysis were related to *Ruminococcus gauvreauii* (35% relative abundance). *R. gauvreauii* was mainly detected in the xylose enrichment and only less than 1% was found in the dried grass and was not detected in the starch waste enrichments. In the clone library, the sequences related to this member were detected in both xylose and starch waste enrichments with 10% and 3% relative abundances with 96–98% and 96% 16S rRNA identity, respectively (Table S4). *Ruminococcus gauvreauii* (CCRI-16110^T^) has been reported not to produce acid from l-xylose or starch [8]. However, in our experiment, we used d-xylose and starch waste in which the composition of xylose and starch were different from the previous study. Based on the 16S rRNA gene sequence identities of our clones (96–98%) to that of *R. gauvreauii* CCRI-16110^T^, we enriched one or more novel species of the genus *Ruminococcus*.

### Isolation and Identification of Fast-Growing Bacteria

The secondary enrichments of dried grass, starch waste and xylose were selected to isolate potential novel species. Twelve isolates were obtained and identified based on 16S rRNA gene sequence analysis which resulted in eight unique isolates (Table S5). One of the isolates, strain Cavy grass 6, is a novel species and it is a facultative anaerobic, fast-growing bacterium that converts cellobiose mainly to lactate [25].

## Conclusion

We investigated the microbial community of faecal samples from two domesticated guinea pigs using MiSeq sequencing analysis and found that 68% of the community could not be classified to genus level. The microbial composition of the two faecal samples was quite different at the genus level (Fig. 1), indicating that despite identical diets for several years, the two guinea pigs each have their own microbiomes.

This was the first study in which guinea pig faecal samples were used for organic acid production and microbial enrichments with various substrates. Acetate was the main organic acid produced from all substrates. *Prevotella* and *Blautia* were the most abundant microbial groups in the secondary enrichments of dried grass, starch waste and xylose. The microbial enrichment strategy is an efficient approach for obtaining novel organic acid-producing bacteria. The microbial diversity analysis of the guinea pig intestine has been reviewed, and many unknown bacteria are waiting to be cultured and characterized. Therefore, guinea pig faecal samples are an interesting source for further microbial exploration and could lead to the isolation of dedicated acetate or lactate producers and/or starch waste, grass or cellulose degraders in the future.

## Electronic supplementary material

Below is the link to the electronic supplementary material.

Supplementary material 1 (PDF 266 KB)
