# Supplementary material for: Microbial Diversity and Organic Acid Production of Guinea Pig Faecal Samples
Source: Curr Microbiol. 2019 Feb 12;76(4):425–34. doi: 10.1007/s00284-019-01630-x (PMC6427046; doi:10.1007/s00284-019-01630-x)
Supplement: Supplementary file 1 — Supplementary material 1 (PDF 266 KB) [file 284_2019_1630_MOESM1_ESM.pdf]

Supplementary material

Microbial diversity and organic acid production of guinea pig faecal samples

Susakul Palakawong Na Ayudthaya[1, 2], Hans van der Oost[1], John van der Oost[1], Daan M. van Vliet[1] and Caroline M. Plugge[1]

[1] Laboratory of Microbiology, Wageningen University & Research, Stippeneng 4, 6708 WE, Wageningen, The Netherlands.

[2] Biodiversity Research Centre, Thailand Institute of Scientific and Technological Research 35 Mu 3, Khlong Ha, Khlong Luang, Pathum Thani 12120 Thailand

Correspondence: Susakul Palakawong Na Ayudthaya

E-mail: susakul@tistr.or.th, Tel. +66 2 577 9027, Fax. +66 2 577 9031

Supplementary table

Table S1. Total number of OTUs detected in each Cavy faecal sample

Sample	Actinobacteria	Archaea	Bacteroidetes	Cyanobacteria	Fibrobacteres	Firmicutes	Proteobacteria	Spirochaetae	Synergistia	Tenericutes	Verrucomicrobia	Unclassified
**Cavy 1**	5	1	13	2	0	16	2	1	1	2	6	1
**Cavy 2**	4	1	13	3	1	31	1	1	0	1	7	1

Table S2. Fermentation profiles in the primary enrichments

Substrates (0.5 % w/v)	Incubation time (days)	Glucose	Products (mmol l^{-1})						
			Acetate	*Butyrate*	*Formate*	*Lactate*	*Propionate*	*Succinate*	*Ethanol*
No substrate	*0*	0	0	0	0	0	0	0	2.2 (± 0.0)
	1	0	0	0	0	0	0	0	2.2 (± 1.7)
	3	0	3.5 (± 0.4)	0	1.3 (± 0.0)	0	0	0.2 (± 0.2)	2.0 (± 0.6)
	7	0	4.7 (± 0.2)	0.7 (± 0.0)	0	0	0	0.3 (± 0.0)	2.8 (± 0.4)
	9	0	4.7 (± 0.4)	0.2 (± 0.3)	0	0	0	0.1 (± 0.2)	0
	14	0	5.0 (± 0.6)	0.2 (± 0.3)	0	0	0	0	1.9 (± 2.7)
Cellulose	*0*	0	0	0	0	0	0	0	0
	1	0	0	0	0	0	0	0	2.5 (± 0.4)
	3	0	3.2 (± 1.0)	0.7 (± 1.0)	0.7 (± 1.0)	0	0	0.2 (± 0.2)	5.6 (± 0.5)
	7	0	11.4 (± 0.4)	2.1 (± 0.5)	0	0	0	7.0 (± 0.8)	0
	9	0	12.0 (± 0.1)	1.9 (± 0.4)	0	0	0	7.9 (± 0.8)	0
	14	0	14.5 (± 0.7)	2.7 (± 0.9)	0	0	0	1.3 (± 1.9)	0
Dried grass	*0*	0.5 (± 0.0)	0	0	0.8 (± 1.2)	0	0	0	1.5 (± 2.2)
	1	0	4.2 (± 0.1)	0.8 (± 0.1)	0	2.1 (± 0.1)	0	0.6 (± 0.1)	2.2 (± 0.1)
	3	0	10.4 (± 0.1)	2.1 (± 0.3)	0	0.3 (± 0.0)	1.3 (± 0.1)	0	0
	7	0	17.5 (± 1.6)	3.3 (± 0.0)	0	0	3.8 (± 0.1)	0	0
	9	0	17.6 (± 0.0)	2.9 (± 0.1)	0	0	4.4 (± 0.3)	0	0
	14	0	21.3 (± 0.4)	3.4 (± 0.0)	0	0	6.6 (± 0.2)	0	0
Glucose	*0*	25.8 (± 1.1)	0	0	0	0	0	0	0
	1	0.7 (± 1.1)	14.9 (± 1.1)	0.7 (± 0.1)	16.5 (± 5.4)	11.0 (± 3.0)	3.0 (± 1.0)	1.7 (± 0.0)	8.9 (± 1.6)
	3	0	15.8 (± 0.6)	5.3 (± 0.9)	16.3 (± 5.4)	0	6.0 (± 0.1)	2.1 (± 0.3)	2.9 (± 2.8)
	7	0	16.4 (± 0.9)	5.6 (± 0.5)	16.7 (± 6.1)	0	7.0 (± 1.8)	1.2 (± 1.8)	2.4 (± 0.8)
	9	0	14.8 (± 0.6)	5.1 (± 0.5)	14.6 (± 4.9)	0	6.8 (± 0.8)	0.6 (± 0.9)	3.2 (± 2.7)
	14	0	14.8 (± 0.7)	5.4 (± 0.1)	14.5 (± 5.5)	0	7.6 (± 0.0)	0	1.8 (± 2.6)
Starch waste	*0*	0	0	0	0	1.5 (± 0.1)	0	0	0.7 (± 1.0)
	1	0.5 (± 0.3)	17.5 (± 1.0)	0.7 (± 0.1)	6.3 (± 0.8)	4.6 (± 0.6)	1.4 (± 1.1)	4.4 (± 0.0)	1.0 (± 1.4)
	3	0	23.3 (± 2.0)	3.0 (± 0.2)	3.4 (± 0.2)	0	8.6 (± 0.2)	0	1.3 (±1.8)
	7	0	22.2 (± 0.8)	3.2 (±0.1)	2.6 (± 0.5)	0	8.4 (±0.1)	0	2.2 (±3.1)
	9	0	22.9 (± 1.9)	3.0 (±0.2)	2.6 (± 0.4)	0	8.8 (±0.3)	0	1.2 (±1.7)
	14	0	24.1 (± 0.9)	3.4 (±0.3)	2.7 (± 0.7)	0	9.8 (±0.4)	0	0
Xylan	*0*	0	0	0	0	0	0	0	2.0 (± 0.8)
	1	0	13.7 (± 0.27)	0	1.4 (± 0.0)	1.2 (± 0.1)	0	0.6 (± 0.1)	0.6 (± 0.9)
	3	0	31.4 (± 6.01)	0.9 (± 0.0)	0	0	2.9 (± 0.0)	2.3 (± 0.4)	0.6 (± 0.9)
	7	0	32.1 (± 0.84)	1.3 (± 0.2)	0	0	4.0 (± 0.6)	2.1 (± 1.4)	0.8 (± 1.2)
	9	0	29.7 (± 3.23)	1.2 (± 0.0)	0	0	5.6 (± 0.9)	0	0.9 (± 1.3)
	14	0	29.9 (± 2.66)	1.3 (± 0.1)	0	0	5.8 (± 0.9)	0	0.8 (± 1.2)
Xylose	*0*	0	0	0	0	0	0	0	1.0 (± 1.4)
	1	0	8.7 (± 0.40)	0.5 (± 0.1)	1.3 (± 1.8)	0.6 (± 0.0)	1.2 (± 0.0)	6.1 (± 0.3)	2.4 (± 0.1)
	3	0	14.5 (± 0.98)	3.9 (± 1.1)	4.0 (± 3.1)	0	3.4 (± 0.5)	10.1 (± 0.1)	1.9 (± 2.7)
	7	0	16.1 (± 1.03)	4.9 (± 0.2)	4.1 (± 3.2)	0	3.2 (± 0.1)	10.6 (± 0.5)	1.1 (± 1.6)
	9	0	16.2 (± 2.37)	3.8 (± 0.2)	3.8 (± 3.2)	0	2.7 (± 0.4)	10.8 (± 0.9)	2.3 (± 0.0)
	14	0	13.6 (± 2.74)	3.7 (± 1.0)	2.6 (± 3.6)	0	7.0 (± 4.7)	4.7 (± 5.9)	1.5 (± 2.1)

Table S3. Fermentation profiles in the secondary enrichments

**Substrates (0.5 % w/v)**	**Incubation time (days)**	**Glucose**	**Products (mmol l^{-1})**						
			Acetate	***Butyrate***	***Formate***	***Lactate***	***Propionate***	***Succinate***	***Ethanol***
**Dried grass**	*5*	0	10.5 (± 1.7)	0.5 (± 0.1)	0	0	0.3 (± 0.4)	0.5 (± 0.7)	1.0 (± 1.4)
**Starch waste**	*5*	0	17.0 (± 1.9)	1.5 (± 0.5)	3.7 (± 5.2)	0.5 (± 0.6)	10.1 (± 1.9)	0	5.0 (± 0.9)
**Xylose**	*5*	0	27.0 (± 19.4)	1.8 (± 0.7)	0	0	2.3 (± 3.2)	10.9 (± 1.4)	0

Table S4. Phylogenetic affiliation and frequency of cloned bacterial 16S rRNA gene amplicons[a] from the secondary enrichments of cavy faecal samples

Enrichment (No. of clones)	Closest cultured relative	No. of clones	Identity (%)	%
Dried grass (36)	*Acetanaerobacterium elongatum*	1	93%	3
	Anaerovorax odorimutans	2	93%	6
	Bacteroides xylanisolvens	2	99%	6
	Blautia marasmi	4	98%	11
	Blautia producta	4	98%	11
	[Clostridium] cochlearium	1	99%	3
	Clostridium fimetarium	1	95%	3
	Prevotella copri	3	91%	8
	Prevotella dentalis	9	92-94%	25
	Prevotella veroralis	9	91-92%	25
Starch waste (37)	*Bacteroides coprosuis*	1	89%	3
	Bacteroides xylanisolvens	1	99%	3
	Blautia marasmi	4	96-98%	11
	Blautia producta	5	98%	14
	Clostridium cochlearium	2	99%	5
	[Clostridium] indolis	2	95-99%	5
	Defluviitalea saccharophila	1	97%	3
	Prevotella bryantii	1	95%	3
	Prevotella copri	1	91%	3
	Prevotella dentalis	10	93-95%	27
	Prevotella histicola	2	91-92%	5
	Prevotella maculosa	1	91%	3
	Prevotella veroralis	4	91%	11
	Ruminococcus gauvreauii	1	96%	3
	Sporanaerobacter acetigenes	1	99%	3
Xylose (20)	*Bacteroides coprosuis*	2	89%	10
	Bacteroides eggerthii	1	89%	5
	Bacteroides xylanisolvens	1	90%	5
	Blautia marasmi	3	98-99%	15
	Blautia producta	1	96%	5
	Clostridium cochlearium	3	99%	15
	Defluviitalea saccharophila	1	93%	5
	Eubacterium oxidoreducens	1	93%	5
	Oscillibacter valericigenes	1	92%	5
	Prevotella dentalis	2	94%	10
	Prevotella veroralis	2	91%	10
	Ruminococcus gauvreauii	2	96-98%	10

[a] 16S rRNA gene sequences were deposited in genbank with the accession numbers LT708382-LT08474.

Table S5. Pure cultures obtained from guinea pig faecal samples

Enrichment	No. of isolates	Isolate name	Cell morphology	16SrRNA Sequence length (bp)	Accession no. of the isolates	Closest cultured relative/strain name	Identity (%)
Dried grass	1	Cavy grass 2	Gram-positive, long rod shaped, non-motile	1270	MF579704	*Clostridium saccharolyticum*	96
	1	Cavy grass 5	Gram-positive, long rod-shaped, non-motile	1252	MF579706	*Clostridium jeddahense*	99
	2	Cavy grass 1, 3	Gram-positive coccus-shaped, non-motile	1270, 1296	MF579703, MF579705	*Streptococcus devriesei*	99, 99
	1	Cavy grass 6*	Gram-positive coccus-shaped, non-motile	1412	LT546457	*Streptococcus caviae*	100
Starch waste (Stw)	1	Stw 2	Gram- positive, rod or irregular shaped, non-motile	1284	MF579708	*Actinomyces succiniciruminis*	100
	1	Stw 1	Gram-positive, long rod shaped, non-motile	1253	MF579707	*Clostridium amygdalinum*	99
	3	Stw 3, 4, 5	Gram-positive rod-shaped, non-motile	1269, 1276, 1271	MF579709, MF579710, MF579711	*Lactobacillus mucosae*	100, 99, 100
	1	Stw 6	Gram-positive coccus shaped, non-motile	1272	MF579712	*Staphylococcus capitis*	99
Xylose	1	Xylose 7	Gram-positive, long rod shaped, non-motile	1255	MF579713	*Clostridium merdae*	98

* Cavy grass 6 has already been validly described as *Streptococcus caviae* by Palakawong Na Ayudthaya, et al. [SR1].

Supplementary figure

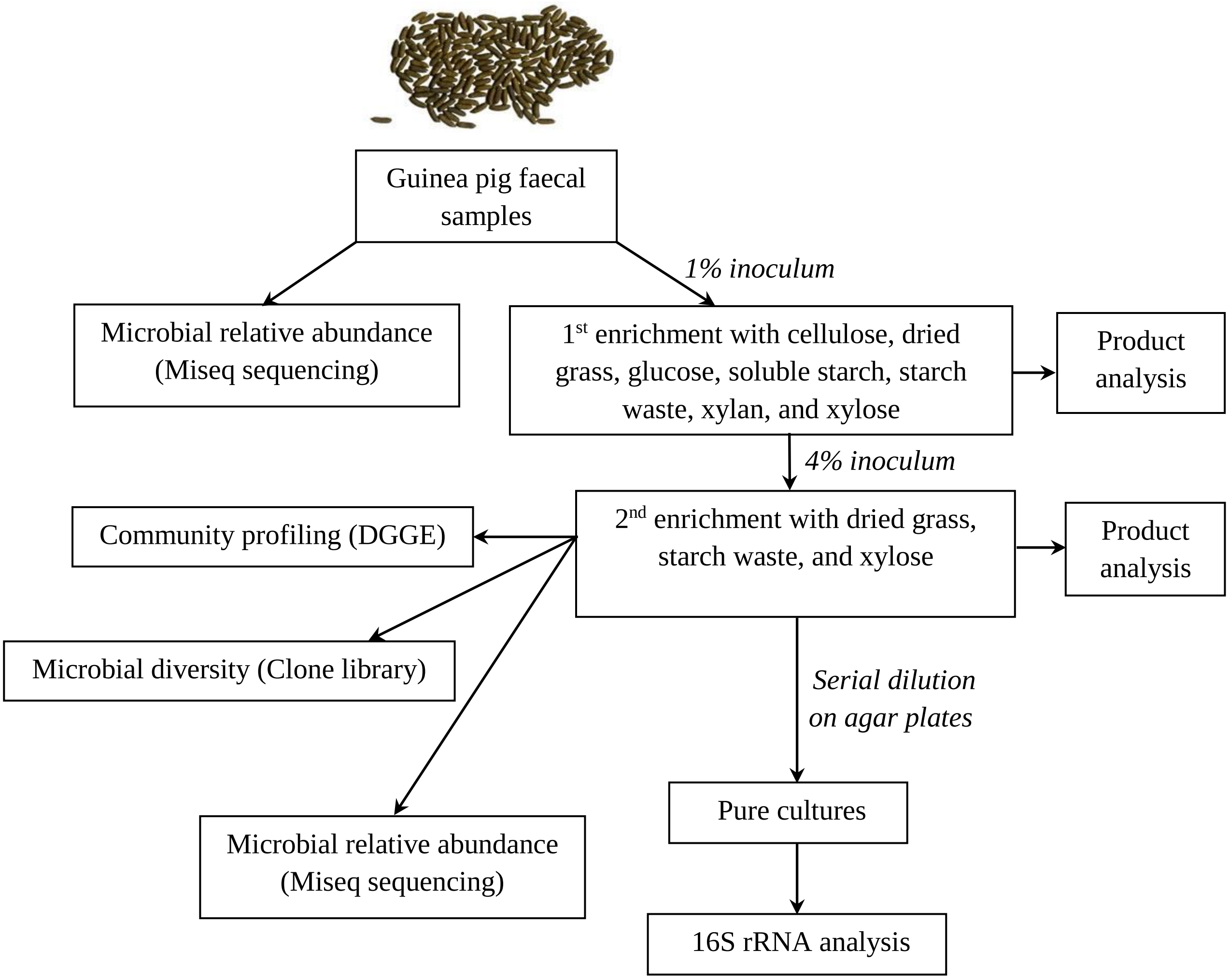

Fig. S1. Schematic overview of the experiments

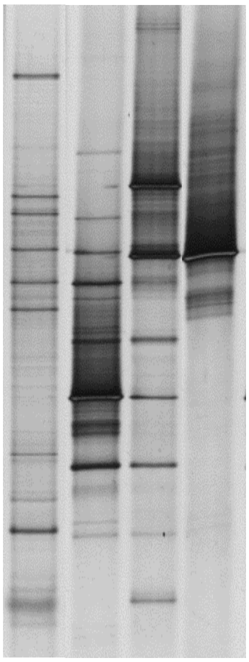

Fig. S2. Bacterial 16S rRNA-DGGE profiles of the secondary enrichments with dried grass (D), starch waste (STW), and xylose (X). M is the marker.

Supplementary References

[SR1] Palakawong Na Ayudthaya S, Hilderink LJ, van der Oost J, de Vos WM, Plugge CM (2017) *Streptococcus caviae* sp. nov., isolated from guinea pig faecal samples. Int J Syst Evol Microbiol 67:1551–1556
